# The Antiarrhythmic Drug Flecainide Enhances Aversion to HCl in Mice

**DOI:** 10.1523/ENEURO.0048-23.2023

**Published:** 2023-09-20

**Authors:** Yuko Kawabata, Shingo Takai, Keisuke Sanematsu, Ryusuke Yoshida, Fuminori Kawabata, Noriatsu Shigemura

**Affiliations:** 1 Section of Oral Neuroscience, Graduate School of Dental Science, Kyushu University, Fukuoka 812-8582, Japan; 2 Research and Development Center for Five-Sense Devices, Kyushu University, Fukuoka 819-0395, Japan; 3 Department of Oral Physiology, Graduate School of Medicine, Dentistry and Pharmaceutical Sciences, Okayama University, Okayama 700-8525, Japan; 4 Physiology of Domestic Animals, Faculty of Agriculture and Life Science, Hirosaki University, Hirosaki 036-8561, Japan; 5 Oral Health/Brain Health/Total Health Research Center, Graduate School of Dental Science, Kyushu University, Fukuoka 812-8582, Japan

**Keywords:** antiarrhythmic drug, flecainide, taste, taste disorder

## Abstract

Drug-induced taste disorders reduce quality of life, but little is known about the molecular mechanisms by which drugs induce taste disturbances. In this study, we investigated the short-term and long-term effects of the antiarrhythmic drug flecainide, which is known to cause taste dysfunction. Analyses of behavioral responses (licking tests) revealed that mice given a single intraperitoneal injection of flecainide exhibited a significant reduction in preference for a sour tastant (HCl) but not for other taste solutions (NaCl, quinine, sucrose, KCl and monopotassium glutamate) when compared with controls. Mice administered a single dose of flecainide also had significantly higher taste nerve responses to HCl but not to other taste solutions. Compared with controls, mice administered flecainide once-daily for 30 d showed a reduced preference for HCl without any changes in the behavioral responses to other taste solutions. The electrophysiological experiments using HEK293T cells transiently expressing otopetrin-1 (Otop1; the mouse sour taste receptor) showed that flecainide did not alter the responses to HCl. Taken together, our results suggest that flecainide specifically enhances the response to HCl in mice during short-term and long-term administration. Although further studies will be needed to elucidate the molecular mechanisms, these findings provide new insights into the pathophysiology of drug-induced taste disorders.

## Significance Statement

Drug-induced taste disorders reduce quality of life and can lead to nutritional disturbances. However, little is known about its molecular mechanisms. We focused on the antiarrhythmic drug flecainide inducing “unpleasant or bad taste” in human patients. Mice administered a single dose of flecainide exhibited a reduced preference for and higher taste nerve responses to HCl, sour tastants specifically. Flecainide had little change in response to HCl in HEK293T cells expressing the sour taste receptor, proton channel otopetrin-1 (Otop1). Our results suggest that flecainide enhances the responses of sour-sensing taste cells to HCl. Although further studies will be needed to elucidate the molecular mechanisms, these findings provide new insights into the pathophysiology of drug-induced taste disorders.

## Introduction

More than 280 different medications are known to cause alterations in taste perception in patients, and these adverse reactions are known as drug-induced taste disorders (Naik et al., 2010). Such drug-induced taste disorders reduce quality of life and can lead to nutritional disturbances, malnutrition, and poor adherence to management plans. However, little is known about the molecular mechanisms that underlie drug-induced taste disorders.

Flecainide is an antiarrhythmic drug known to cause taste dysfunction. Flecainide, a widely used antiarrhythmic drug, prevents supraventricular and ventricular arrhythmias, paroxysmal atrial fibrillation and flutter (Mueller and Baur, 1986). The main antiarrhythmic action of this drug is thought to be because of the inhibition of voltage-gated sodium channels (SCN5A), which slows the conduction of electrical impulses within the heart and prolongs the refractory period of ventricular and atrial myocytes (H. Liu et al., 2002). Flecainide also inhibits cardiac potassium channels (KCNB1) contributing to the delayed rectifier potassium current (Tamargo et al., 2004), which also increases cardiac refractoriness. Additionally, flecainide was reported to block calcium release channel, ryanodine receptor-2 (RyR2), and thereby suppress calcium waves in cardiomyocytes and prevent catecholaminergic polymorphic ventricular tachycardia in mice and humans (Watanabe et al., 2009). A study examining the long-term efficacy of flecainide in the treatment of supraventricular tachycardia found that some patients experienced central nervous system side effects such as visual disturbances (∼20% of cases), nervousness (4%), dizziness (2.4%), and taste disturbances (5%; Neuss, 1985). Furthermore, some patients describe “unpleasant or bad taste” as an adverse reaction of flecainide (Muhiddin et al., 1985; Kreeger and Hammill, 1987). However, the molecular mechanisms underlining the unwanted effects of flecainide on the taste system have not been elucidated.

Taste buds consist of 50–100 taste cells that interact with gustatory nerves. Recent molecular studies have discovered candidate receptors for five basic tastes (Lindemann, 2001; Chandrashekar et al., 2006; Shigemura and Ninomiya, 2016). These receptors are divided into two types: G-protein-coupled receptors (GPCRs) that discriminate sweet, bitter and umami tastes, and channel-type receptors that discriminate salty and sour tastes. The taste receptors for sweet [taste receptor type 1 member 2 (T1R2) and T1R3], bitter [taste receptor type 2 (T2R)], salty [epithelial sodium channel (ENaC)] and sour [otopetrin-1 (Otop1); Tu et al., 2018] are expressed in a distinct subset of cells in taste buds, which suggests that the coding of taste quality may occur at the level of taste cells. Taste cells expressing GPCRs for sweet, bitter or umami use multiple ion channels such as TRP channel subfamily M member 5 (TRPM5), voltage-gated sodium channels (SCN2A, SCN3A, SCN9A) and voltage-gated potassium channels (KCNQ1) in the transduction cascades (Ohmoto et al., 2006; Wang et al., 2009). The other taste cells expressing Otop1, use voltage-gated calcium channels (alpha1A) in addition to SCN2A, and potassium channels (KCNQ1, Kir2.1; Medler et al., 2003; L. Liu et al., 2005; DeFazio et al., 2006; Gao et al., 2009; Ye et al., 2016). Furthermore, L-type voltage-gated calcium channels are thought to interact with RyRs in mouse taste cells (Rebello et al., 2013). These taste type-specific ion channels play important roles in the regulation of distinct taste cell excitability.

We thus explored the pathomechanisms of the adverse reactions of flecainide on the taste system. We investigated the effects of flecainide on the behavioral and neural responses of mice to taste stimuli, the whole cell current of HEK293T cells transiently expressing mouse Otop1. We found that flecainide specifically enhances the sour taste substance HCl responses in mice. The results in Otop1-expressing cells provided no suggestion that flecainide may be involved in a pathway of sour-sensing proton channel, Otop1. A much more extensive set of experiments is required to demonstrate whether Otop1 is involved. The above actions of flecainide may contribute to the taste disorders experienced by patients as an adverse reaction of this antiarrhythmic drug.

## Materials and Methods

### Animals

Mouse husbandry and all mouse experiments were conducted in accordance with the ethical guidelines of Kyushu University and the Rules for Animal Experimentation of Hirosaki University. All experimental protocols and procedures were approved by the Committee for Laboratory Animal Care and Use at Kyushu University (approval no. A19-286-0) and the Animal Research Committee of Hirosaki University (approval no. A18002, A19002 and A19009) and were in accordance with the National Institutes of Health *Guide for the Care and Use of Laboratory Animals*. C57BL/6J mice were purchased from Charles River Laboratories Japan and CLEA Japan. All mice were housed in a 12/12 h light/dark cycle at 23°C and had *ad libitum* access to water and food pellets (CE-2, CLEA Japan). Male mice aged 8–12 weeks were used for all experiments (Takai et al., 2015).

### Taste compounds and drugs

The following taste solutions were used in this study: acetic acid (AA), citric acid (CA), HCl, NaCl (with or without amiloride), KCl, sucrose [with quinine-HCl (QHCl)], QHCl, NH_4_Cl (all purchased from Fujifilm Wako Pure Chemical Corporation) and monopotassium glutamate (MPG; Sigma-Aldrich). All taste solutions were dissolved in distilled water (DW; Yoshida et al., 2010). The following antiarrhythmic drugs were used: amiodarone (A2530, Tokyo Chemical Industry), flecainide (F6777, Sigma-Aldrich) and propafenone (P2301, Tokyo Chemical Industry). All drugs were dissolved in dimethyl sulfoxide (Fujifilm Wako Pure Chemical Corporation). For the behavioral and neural experiments, a stock solution of flecainide was diluted in vehicle (5% glucose) to its final concentration just before administration by intraperitoneal injection. The dose of flecainide administered was determined with reference to the dose for humans stated in the prescribing information.

### Short-term lick test

Details of the procedures used for this test are described in our previous papers (Yoshida et al., 2010; Shigemura et al., 2013; Takai et al., 2015). Each animal was deprived of water for 23 h, placed in the test cage on day 1 of training and then given free access to DW during a 1-h session. During training sessions on days 2–5, the animal was trained to drink DW on an interval schedule that consisted of 10-s periods of presentation of DW alternated with 20-s inter-trial intervals. On day 6, the number of licks for each test stimulus and DW was counted during the first 10 s after the animal’s first lick using a lick meter (Yutaka Electronics). Measurements of the number of licks 30 min after the intraperitoneal injection of drug were made in the following four experimental groups: (1) a single injection of vehicle (5% glucose); (2) a single injection of flecainide (2 mg/kg body weight, equivalent to the maximal daily dose in humans) dissolved in vehicle (Watanabe et al., 2009); (3) repeated administrations of vehicle for 30 d; and (4) repeated administrations of flecainide (2 mg/kg body weight) for 30 d. The measurements were made the day after last administration in the repeated administration groups. On each test day, the first test stimulus given to the animal was DW, and then the following solutions were tested in a randomized order: 1–50 mm AA, 1–30 mm CA, 1–30 mm HCl, 30–1000 mm NaCl (with and without 30 μm amiloride), 10–300 mm KCl, 30–1000 mm sucrose (with 0.1 mm QHCl), 0.003–3 mm QHCl and 1–1000 mm MPG. The mean value of the tastant/DW lick ratio for each test stimulus was calculated for each animal.

### Recording of CT nerve responses

Whole nerve responses to lingual application of tastants were recorded from the CT nerve as described previously (Yoshida et al., 2010; Shigemura et al., 2013; Takai et al., 2015). The trachea of each mouse was canulated under pentobarbital anesthesia (50–60 mg/kg body weight), and the mouse was then fixed in the supine position with a head holder to allow dissection of the CT nerve. The right CT nerve was dissected free from surrounding tissues after removal of the pterygoid muscle and cut at the point of its entry to the bulla. The entire nerve was placed on an Ag/AgCl electrode. An indifferent electrode was placed in nearby tissue. Neural activity was fed into an amplifier (K-1; Iyodenshikagaku) and monitored on an oscilloscope and audio monitor. Whole nerve responses were integrated with a time constant of 1.0 s and recorded on a computer using a PowerLab/sp4 system (ADInstruments). For taste stimulation of the fungiform papillae, the anterior half of the tongue was enclosed in a flow chamber made of silicone rubber. Taste solutions (100 mm NH_4_Cl, 3–50 mm AA, 1–30 mm CA, 1–30 mm HCl, 30–1000 mm NaCl, 100 mm KCl, 30–1000 mm sucrose, 20 mm QHCl and 100 mm MPG) were delivered to each part of the tongue by gravity flow for 30 s. The tongue was washed with DW for ∼1 min between successive stimulations. After a series of control responses had been recorded, each mouse received a single intraperitoneal injection of flecainide (2 mg/kg body weight) dissolved in vehicle (5% glucose). Another series of responses was recorded 15–30 min after flecainide administration, in accordance with a previous study (Watanabe et al., 2009). Only data from stable recordings were used for the analysis. The magnitude of the integrated whole nerve response was measured during 30-s stimulation. The response was averaged over a 20-s period after excluding the data for the initial and final 5-s periods, and this value was normalized to the response to 100 mm NH_4_Cl to account for interanimal variations in the absolute responses. Because the cell type involved in the NH_4_Cl response may be Type III cells (Oka et al., 2013), the NH_4_Cl response was normalized to the baseline, in accordance with a previous study (Vandenbeuch et al, 2013, 2015; Larson et al., 2015, 2020). The baseline-normalized NH_4_Cl response was not significantly altered by flecainide administration.

### Immunohistochemistory

The dissected tongues of each animal (*n* = 3) were fixed in 4% paraformaldehyde (PFA) in PBS for 50 min. After dehydration with sucrose solution (10% for 1 h, 20% for 1 h, 30% for 3 h at 4°C), the frozen block of fixed tissue was embedded in optimal cutting temperature (OCT) compound (Sakura Finetek) and sectioned into 10-μm-thick slices, which were mounted on silane-coated glass slides. Next, sections of the tongue incubated for 1 h in Blocking One solution (Nacalai Tesque) and then incubated overnight at 4°C with the primary antibody against PLCβ2 (1:200; rabbit anti-PLCβ2, Santa Cruz Biotechnology) or CA4 (1:100; goat anti-CA4, R&D Systems). After washing with TNT buffer, the slides were incubated for 2 h with secondary antibody: Alexa Fluor 568 donkey anti-rabbit IgG (Invitrogen) for PLCβ2, and Alexa Fluor 488 donkey anti-goat IgG (Invitrogen) for CA4.

Immunofluorescence of labeled taste cells was observed using a laser scanning microscope (FV-1000, Olympus); images were obtained using Fluoview software (Olympus). To determine the number of cells expressing PLCβ2 and CA4, we counted positive cells in each taste bud in horizontal sections of the circumvallate papillae. Image-ProPlus (version 4.0; Mediacybernetics) was used to exclude artifactual signals; the cells showing a signal density greater than the mean plus two standard deviations of the density in taste cells in the negative control (primary antibodies omitted) were considered positive.

### Construction of mouse Otop1

Total RNA was isolated from mouse kidney, and first-strand cDNA was synthesized using the SuperScript IV First-strand Synthesis System (Thermo Fisher Scientific). The deduced open reading frames (ORFs) of mouse *Otop1* (m*Otop1*) were amplified in two steps by nested-PCR using PrimeSTAR MAX (TaKaRa Bio). Exon-spanning primers were designed based on the m*Otop1* nucleotide sequence in the NCBI database (accession no. NM_172709.3). The PCR products of the ORFs were subcloned into the pcDNA5/FRT mammalian expression vector using the In-Fusion HD Cloning kit (Takara Bio). The entire sequence of m*Otop1* was confirmed using a BigDye Terminator system (Applied Biosystems).

### Electrophysiology

Whole-cell patch-clamp recordings were performed as described previously (Liang et al., 2019). m*Otop1*/pcDNA5/FRT/TO was co-transfected with EGFP/pCAGGS into HEK293T cells using Screen*Fect* A. The standard and acidic bath solutions contained 140 mm NaCl, 5 mm KCl, 10 mm HEPES, 2 mm MgCl_2_, 2 mm CaCl_2_, and 10 mm glucose. The acidic bath solutions contained 5 mm HCl with 15 μm flecainide or DMSO (dimethyl sulfoxide; the vehicle of flecainide). As the human ASIC1a channel in HEK293T cells can be blocked by a pH 6.8 bath solution (Gunthorpe et al., 2001), we adjusted the standard bath solution to pH 6.8 with NaOH. Cells were voltage-clamped at −60 mV using an EPC10 amplifier (HEKA Elektronik). Patch pipettes had resistances between 2 and 5 MΩ, and they were filled with a pipette solution consisting of 140 mm KCl, 5 mm EGTA, and 10 mm HEPES. We adjusted the pipette solution to pH 7.4 with KOH. 5 mm HCl + DMSO in bath solution was pH 3.22 and 5 mm HCl + 15 μm flecainide in bath solution was pH 3.21. There was no difference between the two solutions.

### Statistical analysis

Data are shown as mean ± SD. The short-term lick scores and the CT nerve responses were compared by unpaired *t* test with Bonferroni correction as *post hoc* test preceded by factorial two-way ANOVA. The electrophysiology and the immunohistochemistry were compared by unpaired *t* test. IBM SPSS Statistics (IBM Corp.) was used to perform all calculations.

## Results

### Short-term effects of flecainide on the behavioral responses of mice to taste stimuli

**Figure 1.**
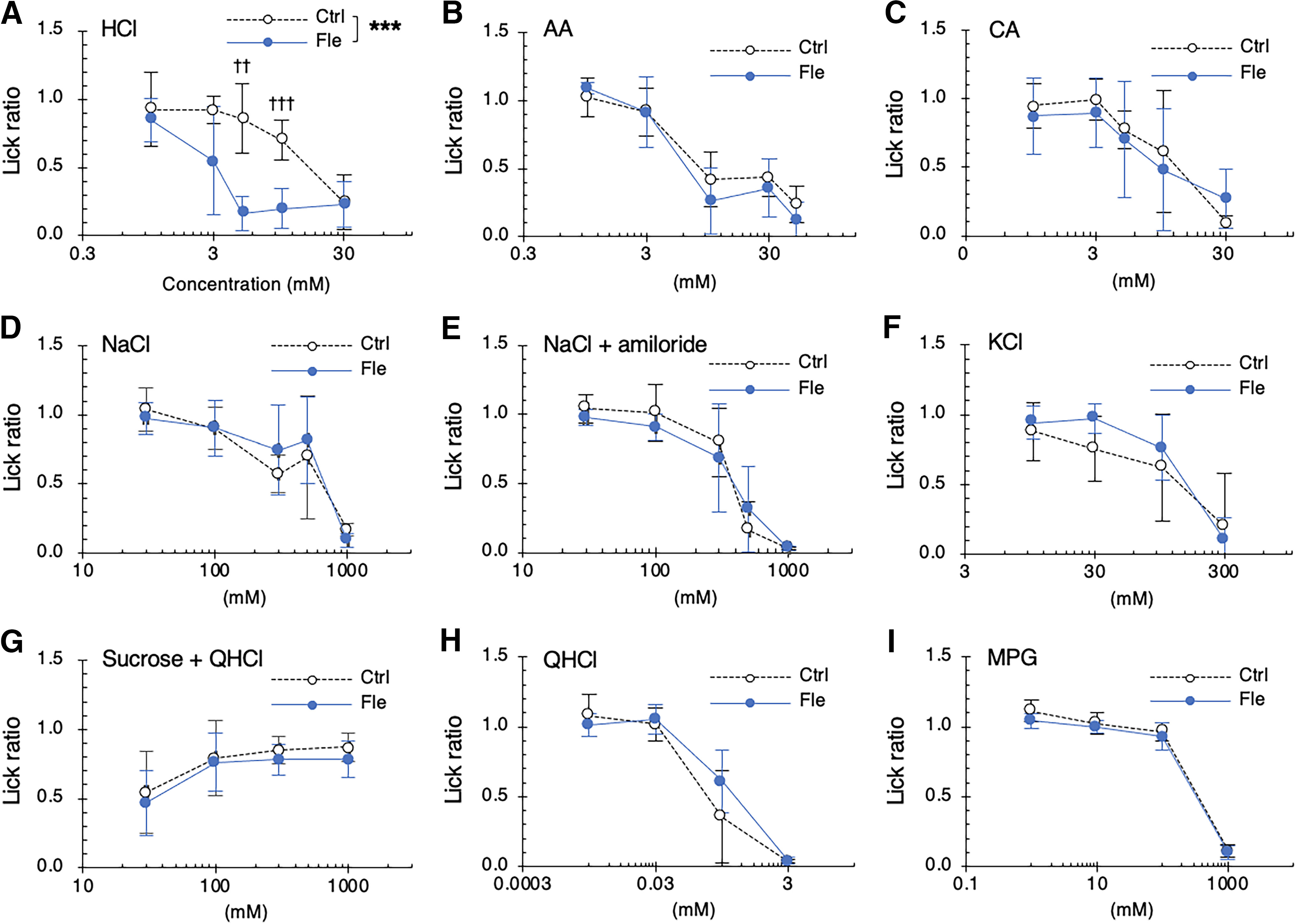
Short-term administration of flecainide enhances the behavioral responses of mice to HCl. Concentration-response relationships for varying concentrations of HCl (***A***), acetic acid (AA; ***B***), citric acid (CA; ***C***), NaCl (***D***), NaCl + 30 μm amiloride (***E***), KCl (***F***), sucrose + 0.1 mm quinine-HCl (QHCl; ***G***), QHCl (***H***) and monopotassium glutamate (MPG; ***I***) 30 min after intraperitoneal injection of vehicle (white symbols) or 2 mg/kg body weight flecainide (Fle, blue symbols). The lick ratio to distilled water is presented as the mean ± SD (*n* = 5–8). ***p <* *0.001 (two-way ANOVA), ^††^*p *<* *0.002, ^†††^*p *<* *0.0002 (unpaired *t* test with Bonferroni correction).

First, we investigated the effects of a single dose of flecainide on the licking behavior of mice in response to taste stimuli. The lick ratio for HCl was significantly lower in mice treated with flecainide than in control mice (*F*_(1,60)_ = 41.383, *p *<* *0.001, ANOVA, effect of flecainide; Fig. 1*A*; Table 1). By contrast, the lick ratios for other taste solutions including weak acids (AA and CA), NaCl, NaCl plus amiloride, KCl, QHCl, sucrose plus QHCl, and MPG were not significantly altered by flecainide (*p *>* *0.05, ANOVA, Fig. 1*B–I*; Table 1).

**Table 1:** Results of statistical analysis for the effect of injection of Fle on the lick ratio (**Fig. 1)**

Figure	Content	Analysis			*p* value
1*A*	Injection (Ctrl vs Fle) × concentration [HCl]	Two-way ANOVA	Injection (Ctrl vs Fle) Concentration Injection × concentration	*F*_(1,60)_ = 41.383 *F*_(4,60)_ = 21.071 *F*_(4,60)_ = 5.777	<0.001 <0.001 0.001
		Unpaired *t* test with Bonferroni correction	Ctrl vs Fle	1 mmol/l HCl 3 mmol/l HCl 5 mmol/l HCl 10 mmol/l HCl 30 mmol/l HCl	0.523 0.023 <0.001 <0.0001 0.869
1*B*	Injection (Ctrl vs Fle) × concentration [AA]	Two-way ANOVA	Injection (Ctrl vs Fle) Concentration Injection × concentration	*F*_(1,38)_ = 1.274 *F*_(4,38)_ = 44.645 *F*_(4,38)_ = 0.614	0.266 <0.001 0.655
1*C*	Injection (Ctrl vs Fle) × concentration [CA]	Two-way ANOVA	Injection (Ctrl vs Fle) Concentration Injection × concentration	*F*_(1,40)_ = 0.209 *F*_(4,40)_ = 9.843 *F*_(4,40)_ = 0.367	0.650 <0.001 0.830
1*D*	Injection (Ctrl vs Fle) × concentration [NaCl]	Two-way ANOVA	Injection (Ctrl vs Fle) Concentration Injection × concentration	*F*_(1,63)_ = 0.411 *F*_(4,63)_ = 38.420 *F*_(4,63)_ = 0.970	0.524 <0.001 0.431
1*E*	Injection (Ctrl vs Fle) × concentration [NaCl + amiloride]	Two-way ANOVA	Injection (Ctrl vs Fle) Concentration Injection × concentration	*F*_(1,45)_ = 0.228 *F*_(4,45)_ = 56.078 *F*_(4,45)_ = 0.833	0.635 <0.001 0.512
1*F*	Injection (Ctrl vs Fle) × concentration [KCl]	Two-way ANOVA	Injection (Ctrl vs Fle) Concentration Injection × concentration	*F*_(1,42)_ = 1.243 *F*_(3,42)_ = 20.811 *F*_(3,42)_ = 0.753	0.271 <0.001 0.527
1*G*	Injection (Ctrl vs Fle) × concentration [Sucrose + QHCl]	Two-way ANOVA	Injection (Ctrl vs Fle) Concentration Injection × concentration	*F*_(1,52)_ = 1.634 *F*_(3,52)_ = 8.751 *F*_(3,52)_ = 0.060	0.207 <0.001 0.980
1*H*	Injection (Ctrl vs Fle) × concentration [QHCl]	Two-way ANOVA	Injection (Ctrl vs Fle) Concentration Injection × concentration	*F*_(1,37)_ = 1.271 *F*_(3,37)_ = 91.459 *F*_(3,37)_ = 2.004	0.267 <0.001 0.130
1*I*	Injection (Ctrl vs Fle) × concentration [MPG]	Two-way ANOVA	Injection (Ctrl vs Fle) Concentration Injection × concentration	*F*_(1,36)_ = 3.033 *F*_(3,36)_ = 454.823 *F*_(3,36)_ = 0.378	0.090 <0.001 0.769

### Short-term effects of flecainide on the gustatory nerve responses to taste stimuli in mice

**Figure 2.**
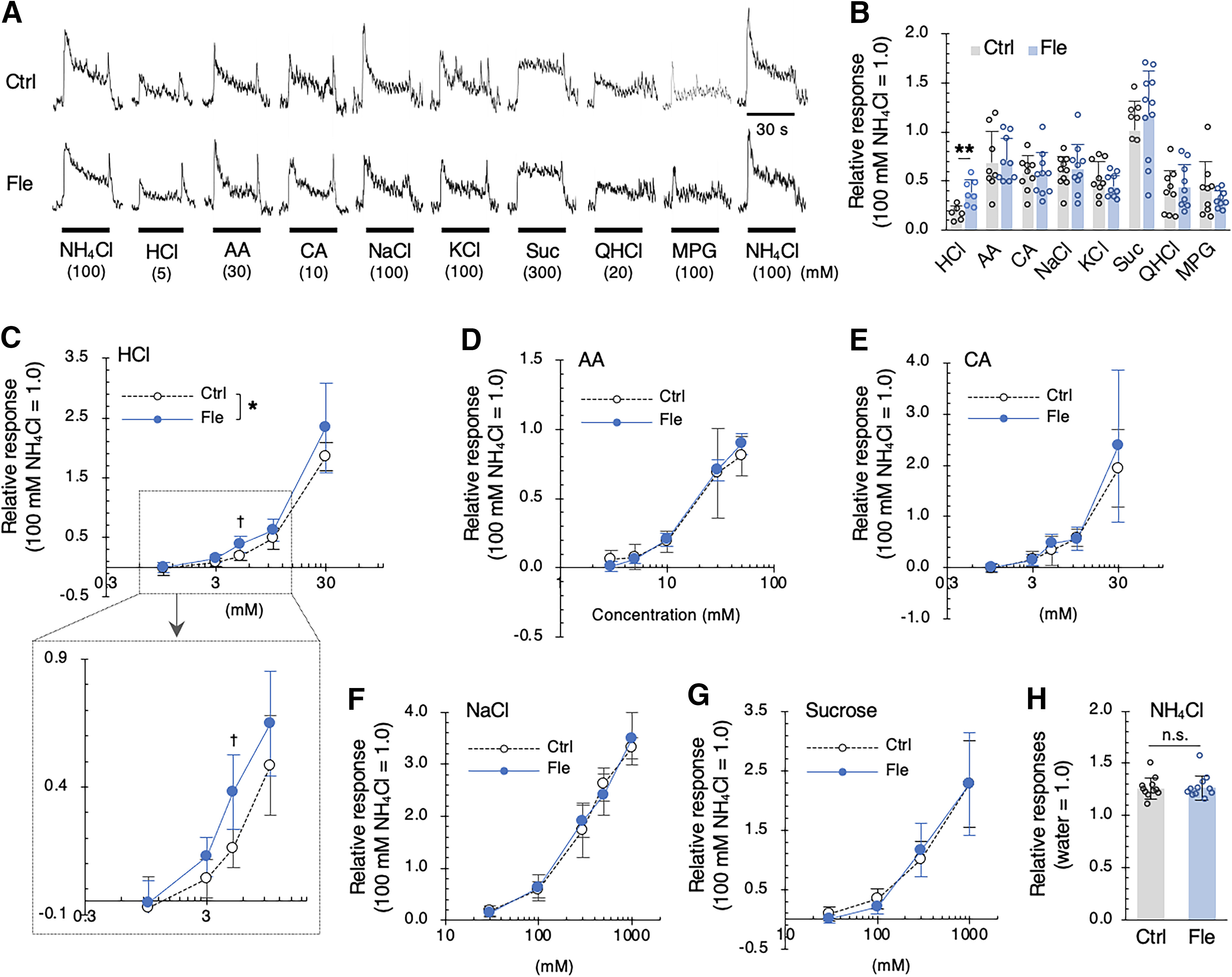
Flecainide enhances corda tympani (CT) nerve responses to HCl in mice. ***A***, Representative examples of CT nerve responses to various taste solutions obtained after the intraperitoneal injection of vehicle (Ctrl, upper traces) or 2 mg/kg body weight flecainide (Fle, lower traces). ***B***, CT nerve responses (normalized to that for 100 mm NH_4_Cl) to sour [5 mm HCl; 30 mm acetic acid (AA); 10 mm citric acid (CA)], salty (100 mm NaCl; 100 mm KCl), sweet [300 mm sucrose (Suc)], bitter [20 mm quinine-HCl (QHCl)] and umami [100 mm monopotassium glutamate (MPG)] compounds recorded 15–30 min after the administration of vehicle (Ctrl, gray bars) or 2 mg/kg body weight flecainide (Fle, blue bars). Data are presented as the mean ± SD (*n* = 6–11). **p <* *0.01 (unpaired *t* test). (***C–G***) Concentration-dependent responses to HCl (***C***) AA (***D***), CA (***E***), NaCl (***F***), and sucrose (***G***) obtained 15–30 min after the administration of vehicle (Ctrl, white symbols) or 2 mg/kg body weight flecainide (Fle, blue symbols). Data are presented as the mean ± SD (*n* = 4–11). *p <* *0.05 (two-way ANOVA), ^†^*p *<* *0.01 (unpaired *t* test with Bonferroni correction). ***H***, CT nerve responses (normalized to that for distilled water) to 100 mm NH_4_Cl recorded 15–30 min after the administration of vehicle (Ctrl, gray bar) or 2 mg/kg body weight flecainide (Fle, blue bar). Data are presented as the mean ± SD (*n* = 11). *p *=* *0.35 (unpaired *t* test), n.s. = no significance.

Next, we investigated the effects of a single dose of flecainide on the gustatory nerve responses to various taste stimuli in mice. We focused on the response of the chorda tympani (CT) nerve, which innervates the anterior part of the tongue responsible for sour taste discrimination (Teng et al., 2019; Zhang et al., 2019). The CT nerve response to HCl was significantly greater in flecainide-treated mice than in control mice, and there was a significant difference by *post hoc* test only at 5 mm HCl but not at the other concentrations tested; that is, there was an overall effect of injection, and the strongest difference was at 5 mm [*p *<* *0.01, *t* test (Fig. 2*B*,*C*), and *F*_(1,51)_ = 5.658, *p *=* *0.021, ANOVA, effect of flecainide (Fig. 2*C*); Table 2]. What is more, the increased responses to 5 mm HCl after flecainide appeared because of a prolongation of the response, rather than an increase in peak. By contrast, flecainide was without significant effect on the CT nerve responses to other taste solutions such as AA, CA, NaCl, KCl, sucrose, QHCl, and MPG (*p *>* *0.05, *t* test or ANOVA; Fig. 2*A*,*B*,*D–G*; Table 2). Furthermore, flecainide had little effect on the NH_4_Cl responses (*p *=* *0.35, *t* test; Fig. 2*H*).

**Table 2:** Results of statistical analysis for the effect of injection of Fle on the CT nerve response in mice (**Fig. 2)**

Figure	Content	Analysis			*p* value
2*B*	Injection (Ctrl vs Fle)	Unpaired *t* test	Ctrl vs Fle	5 mmol/l HCl 30 mmol/l AA 10 mmol/l CA 100 mmol/l NaCl 100 mmol/l KCl 300 mmol/l Suc 20 mmol/l QHCl 100 mmol/l MPG	0.007 0.873 0.816 0.715 0.337 0.391 0.583 0.280
2*C*	Injection (Ctrl vs Fle) × concentration [HCl]	Two-way ANOVA	Injection (Ctrl vs Fle) Concentration Injection × concentration	*F*_(1,51)_ = 5.658 *F*_(4,51)_ = 101.458 *F*_(4,51)_ = 1.188	0.021 <0.001 0.327
		Unpaired *t* test with Bonferroni correction	Ctrl vs Fle	1 mmol/l HCl 3 mmol/l HCl 5 mmol/l HCl 10 mmol/l HCl 30 mmol/l HCl	0.735 0.066 0.007 0.094 0.200
2*D*	Injection (Ctrl vs Fle) × concentration [AA]	Two-way ANOVA	Injection (Ctrl vs Fle) Concentration Injection × concentration	*F*_(1,50)_ = 0.048 *F*_(4,50)_ = 48.276 *F*_(4,50)_ = 0.207	0.828 <0.001 0.933
2*E*	Injection (Ctrl vs Fle) × concentration [CA]	Two-way ANOVA	Injection (Ctrl vs Fle) Concentration Injection × concentration	*F*_(1,61)_ = 0.657 *F*_(4,61)_ = 31.290 *F*_(4,61)_ = 0.397	0.421 <0.001 0.810
2*F*	Injection (Ctrl vs Fle) × concentration [NaCl]	Two-way ANOVA	Injection (Ctrl vs Fle) Concentration Injection × concentration	*F*_(1,62)_ = 0.187 *F*_(4,62)_ = 249.082 *F*_(4,62)_ = 0.802	0.667 <0.001 0.529
2*G*	Injection (Ctrl vs Fle) × concentration [Sucrose]	Two-way ANOVA	Injection (Ctrl vs Fle) Concentration Injection × concentration	*F*_(1,54)_ = 0.024 *F*_(3,54)_ = 63.015 *F*_(3,54)_ = 0.330	0.876 <0.001 0.804
2*H*	Injection (Ctrl vs Fle)	Unpaired *t* test	Ctrl vs Fle	100 mmol/l NH_4_Cl	0.353

### Long-term effects of flecainide on the behavioral responses of mice to taste stimuli

**Figure 3.**
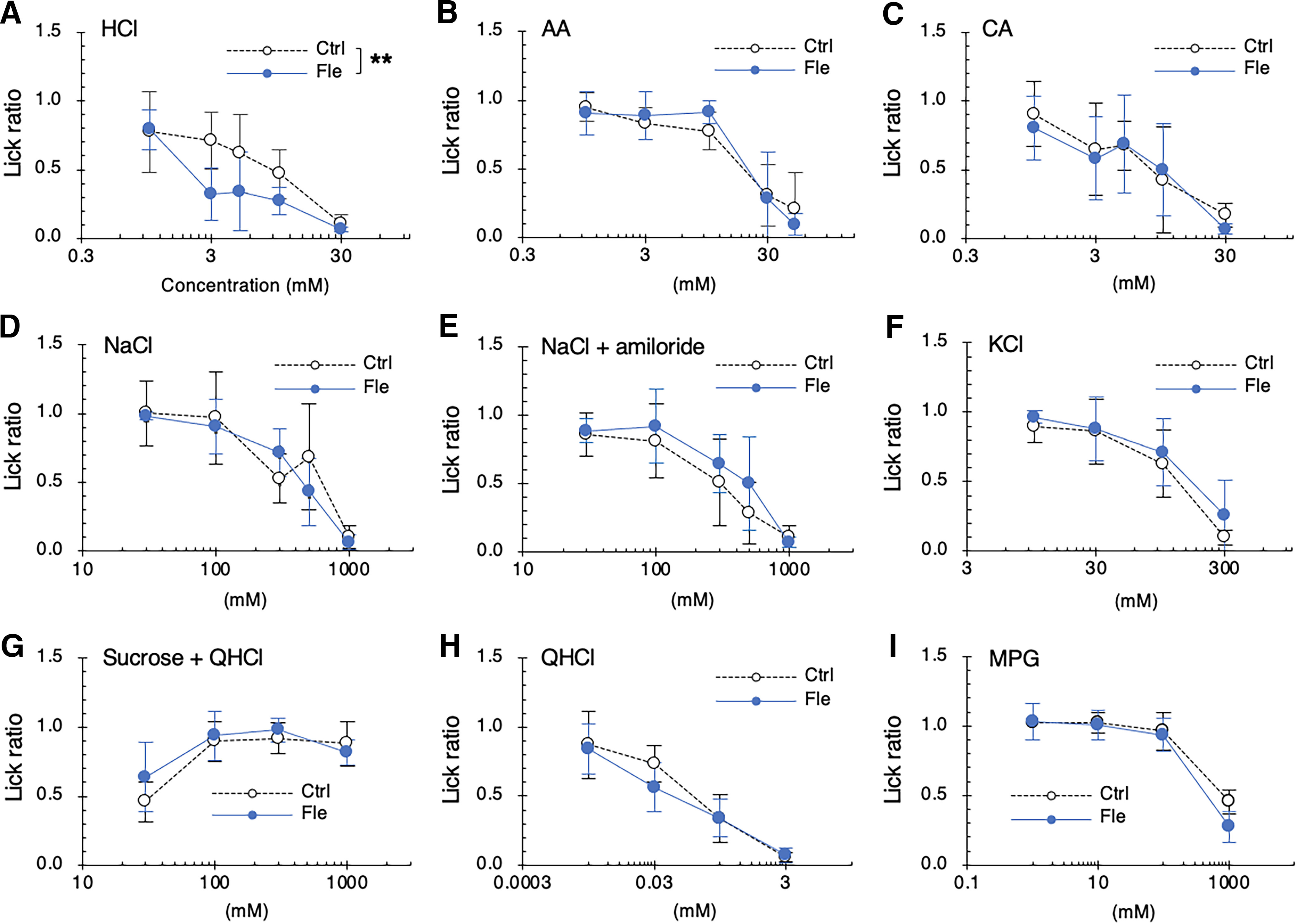
Long-term administration of flecainide enhances the behavioral responses of mice to HCl. Concentration-response relationships for varying concentrations of HCl (***A***), acetic acid (AA; ***B***), and citric acid (CA; ***C***), NaCl (***D***), NaCl + 30 μm amiloride (***E***), KCl (***F***), sucrose + 0.1 mm quinine-HCl (QHCl; ***G***), QHCl (***H***) and monopotassium glutamate (MPG; ***I***) obtained after daily intraperitoneal injections of vehicle (Ctrl, white symbols) or 2 mg/kg body weight flecainide (Fle, blue symbols) for 30 d. The lick ratio to distilled water is presented as the mean ± SD (*n* = 5). **p <* *0.01 (two-way ANOVA).

**Figure 4.**
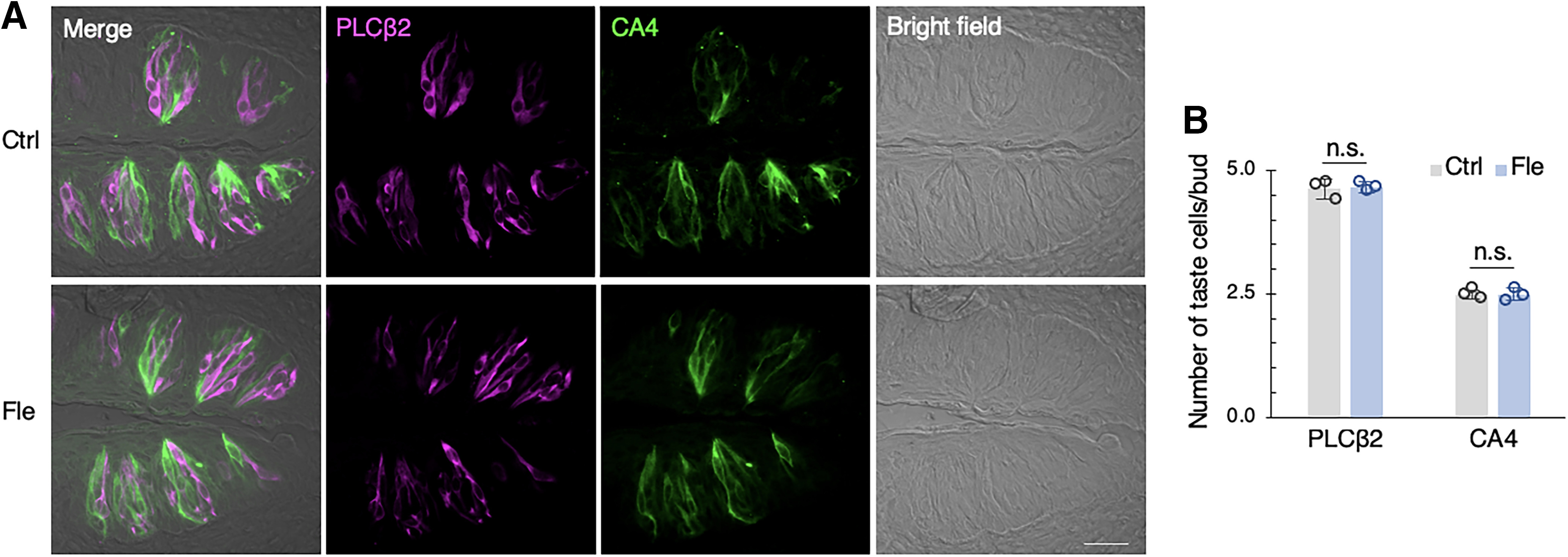
Effects of flecainide on mouse taste bud cells. ***A***, Expression of phospholipase C-β 2 (PLCβ2, a bitter/sweet/umami transduction molecule; magenta), and carbonic anhydrase-4 (CA4, a sour taste sensitive cell marker; green) in the circumvallate papillae after daily intraperitoneal injections of vehicle (Ctrl) or 2 mg/kg body weight flecainide (Fle) for 30 d. Scale bar: 50 μm. ***B***, Quantitation of the number of immunoreactive taste cells per taste bud. Data are expressed as the mean ± SD; *n* = 60–83 taste buds, each (*n* = 3 mice). n.s. = no significance.

We evaluated the long-term effects of flecainide on the behavioral responses to taste stimuli in mice given repeated intraperitoneal injections of flecainide for 30 d. The lick ratio for HCl was significantly lower in flecainide-treated mice than in control mice (*F*_(1,40)_ = 9.977, *p *=* *0.003, ANOVA, effect of flecainide; Fig. 3*A*; Table 3), whereas the lick ratios for other taste solutions (such as NaCl, NaCl plus amiloride, KCl, sucrose plus QHCl, QHCl, MPG, AA, and CA) were not affected by flecainide (*p *>* *0.05, ANOVA; Fig. 3*B–I*; Table 3). No change in the number of Type II and Type III taste cells was observed after 30 d of treatment with flecainide (Fig. 4*A*,*B*; Table 4).

**Table 3:** Results of statistical analysis for the effect of injection of Fle on the lick ratio in mice (**Fig. 3)**

Figure	Content	Analysis			*p* value
3*A*	Injection (Ctrl vs Fle) × concentration [HCl]	Two-way ANOVA	Injection (Ctrl vs Fle) Concentration Injection × concentration	*F*_(1,40)_ = 9.977 *F*_(4,40)_ = 16.123 *F*_(4,40)_ = 1.700	0.003 <0.001 0.169
		Unpaired *t* test with Bonferroni correction	Ctrl vs Fle	1 mmol/l HCl 3 mmol/l HCl 5 mmol/l HCl 10 mmol/l HCl 30 mmol/l HCl	0.909 0.016 0.164 0.062 0.126
3*B*	Injection (Ctrl vs Fle) × concentration [AA]	Two-way ANOVA	Injection (Ctrl vs Fle) Concentration Injection × concentration	*F*_(1,40)_ = 0.006 *F*_(4,40)_ = 37.780 *F*_(4,40)_ = 0.703	0.941 <0.001 0.595
3*C*	Injection (Ctrl vs Fle) × concentration [CA]	Two-way ANOVA	Injection (Ctrl vs Fle) Concentration Injection × concentration	*F*_(1,40)_ = 0.233 *F*_(4,40)_ = 10.386 *F*_(4,40)_ = 0.206	0.632 <0.001 0.933
3*D*	Injection (Ctrl vs Fle) × concentration [NaCl]	Two-way ANOVA	Injection (Ctrl vs Fle) Concentration Injection × concentration	*F*_(1,40)_ = 0.666 *F*_(4,40)_ = 53.723 *F*_(4,40)_ = 2.525	0.419 <0.001 0.056
3*E*	Injection (Ctrl vs Fle) × concentration [NaCl + amiloride]	Two-way ANOVA	Injection (Ctrl vs Fle) Concentration Injection × concentration	*F*_(1,40)_ = 1.929 *F*_(4,40)_ = 21.719 *F*_(4,40)_ = 0.488	0.173 <0.001 0.745
3*F*	Injection (Ctrl vs Fle) × concentration [KCl]	Two-way ANOVA	Injection (Ctrl vs Fle) Concentration Injection × concentration	*F*_(1,32)_ = 1.754 *F*_(3,32)_ = 30.940 *F*_(3,32)_ = 0.219	0.195 <0.001 0.883
3*G*	Injection (Ctrl vs Fle) × concentration [Sucrose + QHCl]	Two-way ANOVA	Injection (Ctrl vs Fle) Concentration Injection × concentration	*F*_(1,32)_ = 1.210 *F*_(3,32_ = 14.212 *F*_(3,32)_ = 1.062	0.279 <0.001 0.379
3*H*	Injection (Ctrl vs Fle) × concentration [QHCl]	Two-way ANOVA	Injection (Ctrl vs Fle) Concentration Injection × concentration	*F*_(1,32)_ = 0.855 *F*_(3,32)_ = 49.522 *F*_(3,32)_ = 0.757	0.362 <0.001 0.526
3*I*	Injection (Ctrl vs Fle) × concentration [MPG]	Two-way ANOVA	Injection (Ctrl vs Fle) Concentration Injection × concentration	*F*_(1,32)_ = 1.382 *F*_(3,32)_ = 48.730 *F*_(3,32)_ = 0.936	0.248 <0.001 0.435

**Table 4:** Results of statistical analysis for the effect of Fle on mouse taste bud cells (**Fig. 4)**

Figure	Content	Analysis			*p* value
4*B*	Taste cells (Ctrl vs Fle)	Unpaired *t* test	Ctrl vs Fle	PLCβ2^+^ cells CA4^+^ cells	0.75 0.97

### Effects of flecainide on the responses of HEK293T cells expressing Otop1

**Figure 5.**
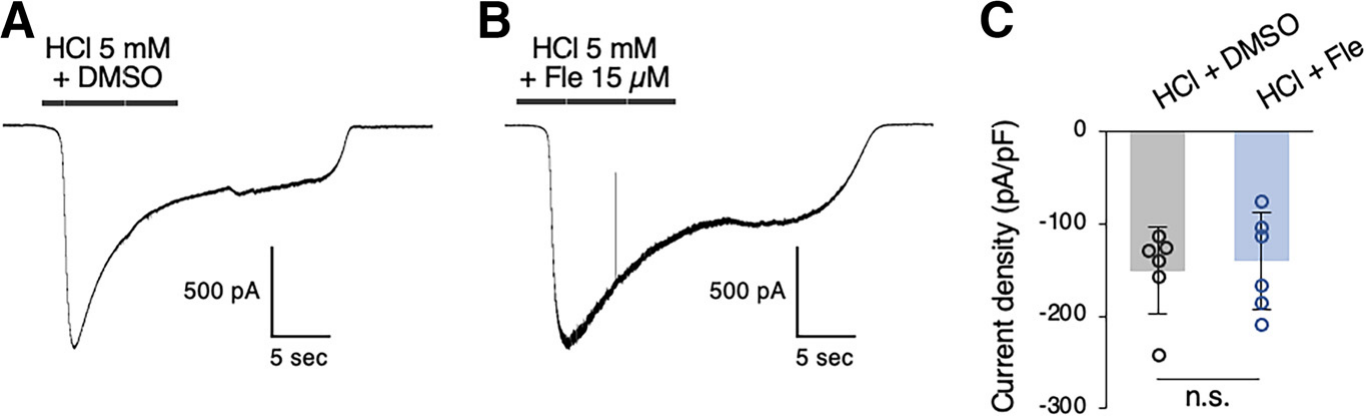
The typical currents of Otop1 by 5 mm HCl with DMSO (the vehicle of flecainide; ***A***) or 5 mm HCl with 15 μm flecainide (***B***) are shown in whole-cell patch clamp tests at −60 mV. ***C***, The comparison of peak current densities of Otop1 between the stimuli of the 5 mm HCl with DMSO and 5 mm HCl with 15 μm flecainide (mean ± SD, *n* = 6 cells). n.s. = no significance.

Our subsequent experiments explored whether the Otop1 channel, a sour taste receptor (Tu et al., 2018; Teng et al., 2019; Zhang et al., 2019), might be involved in the effects of flecainide on the behavioral and neural responses to HCl. We used a m*Otop1*/pcDNA5 construct (Table 5) to generate HEK293T cells transiently expressing mOtop1 protein and analyzed the effects of flecainide on Otop1-expressing cells using whole-cell patch clamp test. The representative currents of Otop1 by 5 mm HCl with DMSO (the vehicle of flecainide) or 5 mm HCl with 15 μm flecainide were shown in Figure 5*A*,*B*, respectively. Although the peak current densities were not different between two stimuli (Fig. 5*C*; Table 6). These results of the present simplified experiments suggest that flecainide does not directly affect the channel activities of Otop1, although the involvement of Otop1 needs to be discussed based on further extensive experiments.

**Table 5:** Primers for construction of mOtop1

	Forward primer	Reverse primer	Product size
1st PCR	GGGGACCAGACTGGAAGATG	GGCAACTCCAGACAGTCAGA	1954
2nd PCR	GTTTAAACTTAAGCTTGGGGA CCAGACTGGAAGATG	CAGCGGGTTTAAACGGGCCCGGCAAC TCCAGACAGTCAGA	1990

**Table 6:** Results of statistical analysis for the effect of application of Fle on the electrophysiology of mOtop1-expressing HEK293T cells (**Fig. 5)**

Figure	Content	Analysis		*p* value
5*C*	Current density (DMSO vs Fle)	Unpaired *t* test	DMSO vs Fle	0.948

## Discussion

The aim of this study was to provide insights into the pathomechanisms underlying flecainide-induced taste disorders. Flecainide is known to cause taste dysfunction as an adverse reaction (Muhiddin et al., 1985; Kreeger and Hammill, 1987), but little is elucidated about the symptoms, and mechanisms. Our *in vivo* experiments were the first to characterize that the administration of flecainide exhibited a reduced preference for and higher taste nerve responses to HCl, sour tastant specifically. However, in the experiments with HEK293T cells expressing mOtop1, we could not observe the effects of flecainide on the response to HCl in HEK293T cells. This discrepancy may be because of the possibility that the enhancement effect of flecainide on HCl is mediated by other sour sensory signals rather than Otop1 in sour taste cells.

Multiple sour taste receptor candidates have been proposed such as acid-sensing ion channels (ASICs; Ugawa et al., 2003), hyperpolarization-activated cyclic nucleotide-gated potassium channels (HCNs; Stevens et al., 2001), potassium channels (Lin et al., 2004; Richter et al., 2004), polycystic kidney disease 2L1 (PKD2L1) and PKD1L3 heteromers (Huang et al., 2006; Ishimaru et al., 2006; Lopezjimenez et al., 2006), and Otop1 (Tu et al., 2018). Recently, it was reported that knock-out of Otop1 abolished sour taste responses from mouse sour-sensing taste receptor cells (Teng et al., 2019). The mice engineered to express Otop1 in sweet taste receptor cells possessed taste cells that responded to both sweet and sour stimuli (Zhang et al., 2019). These findings indicated that Otop1 plays a central role in the perception of sour taste in mice. Flecainide acts as a blocker for several voltage-gated K^+^ channels (Kv), such as hERG (Kv11.1, encoded by *KCNH2*) at clinically relevant concentrations in whole cell voltage clamp recordings of hERG current made from an HEK293 cell line stably expressing hERG (Paul et al., 2002). The previous histologic and transcriptome analyses showed hERG is expressed in sour taste cells (Ohmoto et al., 2006; Ren et al., 2017), thus blockade of hERG by flecainide could increase the excitability of sour cells. In the cardiac inwardly rectifying current, IK_1_, mainly Kir2.1 (*KCNJ2*, encoded in inwardly rectifying potassium channels), flecainide also has a putative differential effect of blocking atrial IK_1_ and increasing ventricular IK_1_ (Caballero et al., 2010). These differences depend on the variations in expression profiles of the other IK_1_ components, Kir2.2 and Kir2.3, between animal species or tissues (Caballero et al., 2010). Kir2.1 is a key component of sour taste transduction by generating response in sour-responsive cells by blocking resting K^+^ currents because of intracellular acidification (Ye et al., 2016). It is possible that flecainide also blocks Kir2.1 in sour cells, resulting in increased cell excitation.

The somatosensory inputs via trigeminal nerve are supposed to be one of the components of the remaining acid responses in Otop1-KO mice (Teng et al., 2019). Proton stimulates TRPV1, a capsaicin, protons, and heat-sensitive nonselective cation channel, in trigeminal neurons (L. Liu and Simon, 2000). According to the previous papers, TRPV1 function (potentiation of capsaicin responses and development of thermal hypersensitivity) was attenuated in P2Y2-KO mice (Malin et al., 2008), and P2Y2-induced hyperalgesia is not observed in TRPV1-KO animals, indicating a functional interaction between TRPV1 and P2Y2 (Moriyama et al., 2003). The activation of P2Y2 downregulate Kv4.2 expression, encoded by *KCND2*, a voltage-gated potassium channel which expressed in trigeminal neurons, results in the reduced current density and the enhanced neuronal excitability in rat trigeminal neurons (Li et al., 2014). Flecainide is reported as a blocker for Kv4.2 in somatosensory neurons (Caballero et al., 2003), thus, an application of flecainide may inhibit Kv4.2 in trigeminal neurons expressing both TRPV1 and P2Y2, and enhance acid sensation via TRPV1. This enhanced sensation in somatosensory neurons may be involved in the increased behavioral aversion to HCl in flecainide-treated animals, in concert with the activation of sour taste cells. In addition, the potentiation of proton sensing may be a possible explanation why changes were observed only in low pH strong acid (HCl) but not for weak acids (AA and CA) in our electrophysiological and behavioral experiments (Table 7).

**Table 7.** The pH value of each acid in the short-term lick test (**Figs. 1 and 3)**

Concentration (mm)		pH
HCl	1 3 5 10 30	3.15 2.64 2.37 2.08 1.63
AA	1 3 10 30 50	3.90 3.71 3.43 3.17 3.04
CA	1 3 5 10 30	3.26 3.05 2.80 2.64 2.43

Various modulators of sour taste have been reported previously. For example, noradrenaline was found to significantly reduce the sour taste threshold by 22% and the bitter taste threshold by 39% in healthy humans (Heath et al., 2006). Mice lacking the receptor for ghrelin, an orexigenic hormone, showed reduced behavioral responses to sour and salty tasting compounds (Shin et al., 2010). Furthermore, mice lacking the receptor for glucagon-like peptide-1, an incretin hormone that stimulates insulin secretion, exhibited reduced sweet taste sensitivity and enhanced citric acid taste sensitivity in behavioral assays (Shin et al., 2008). Thus, although multiple factors have been found to modulate sour taste sensitivity in humans and mice, they were not specific for the sour taste modality. This may be because of nonspecific expression of their cognate receptors in the taste buds. In the present study, flecainide specifically enhanced sour taste without any effects on the other basic taste responses. This is the first finding that sour taste sensitivity is enhanced selectively by the application of a specified substance.

In summary, we report the taste alteration that occurs as an adverse reaction of flecainide, a widely used arrhythmic drug. Our experiments revealed that flecainide specifically enhances the response to HCl, sour tastant in mice during short-term and long-term administration without causing histologic changes. Although further studies will be needed to elucidate the molecular mechanisms, our findings contribute new insights into the understanding of the pathophysiology on drug-induced taste disorders.

## Synthesis

Reviewing Editor: Matthew Grubb, King’s College London Decisions are customarily a result of the Reviewing Editor and the peer reviewers coming together and discussing their recommendations until a consensus is reached. When revisions are invited, a fact-based synthesis statement explaining their decision and outlining what is needed to prepare a revision will be listed below. The following reviewer(s) agreed to reveal their identity: Peihua Jiang. Note: If this manuscript was transferred from JNeurosci and a decision was made to accept the manuscript without peer review, a brief statement to this effect will instead be what is listed below.

Reviewer 1:

This manuscript describes that sour taste can be enhanced by a medicine via modulating the activity of Otop1. If it stands, this would advance our understanding of taste transduction.

Kawabata et al. performed a series of experiments to determine whether flecainide enhances the activity of Otop1, therefore, sour taste. The manuscript is easy to follow. Yet, their conclusion could be further strengthened by providing additional data. Below are my comments:

For Fig. 1, the pH value should be provided for each concentration of acids, including HCl (panel A), AA (panel B), CA (panel C), as Otop1 is a proton channel.

Data provided in Fig. 2 is less convincing, giving the huge variations in the preference ratio among mice in the control group (from strong preference to strong avoidance). The sample size with adequate power may matter here. There is a similar issue for the data in Fig .3C (big error bars).

For Fig. 4, the pH value should be provided for each concentration of acids. Based on my understanding, the authors used HBSS buffer supplemented with 5x test solutions to simulate cells expressing Otop1 or mock transfected cells. Checking pH values of these solutions would be important to interpret data. For comparison of strong and weak acids, solutions with the same pH value should be used.

For Fig.5, only one concentration of HCl was tested at presumably extremely low pH. In previous reports from the Liman lab, they showed that proton currents were evoked by rapidly lowering the pH and the Otop1 channel was closed rapidly after pH returned to 7.4. It makes more sense to include a few more data points (pH lowering to 6, 5.5, 5.0) to see if flecainide indeed affect the decay time.

Reviewer 2

This manuscript reports effects of the antiarrhythmic drug flecainide on taste behavior and physiology in mice. The authors show quite remarkably that a single IP injection of flecainide causes a dramatic change in licking behavior in response to HCl (it is aversive at a lower concentration), but not other sour or non-sour taste stimuli. Gustatory nerve recording showed a small increase in responses to HCl but not other sour or non-sour taste stimuli. These data, while interesting, are not consistent with a role for Otop1 in mediating the effect of flecainide on sour taste, as Otop1 has been shown to mediate responses to all acid stimuli tested in this manuscript (HCl, acetic acid and citric acid). Nonetheless, the authors test for a direct effect of flecainide on Otop1 expressed in HEK 293 cells. They report an increase in the magnitude of the voltage response as measured with voltage imaging in cells expressing Otop1 channels exposed to flecainide. However, this is not substantiated by patch clamp recording, where the authors observe no change in the magnitude of the responses, and instead observe a change in the kinetics of the currents. As described below, changes in voltage in imaging experiments are not reliable because there is no control for the resting voltage and if this is changed, as it very well could be by flecainide, the magnitude of the response evoked by the acid stimulus may change. Similarly, the kinetics of Otop1 currents is not likely to be an inherent feature of the channels, but instead may reflect pH buffering of the cells, which could be affected by flecainide. Thus, while the initial behavioral observation is interesting, and appears to be well substantiated, the evidence points, in my view, to a mechanism that is Otop1-independent. I believe if framed as such, this may nonetheless be a valuable contribution to the literature.

Major concerns.

1. I am concerned that the experimental results may be robust, but that the interpretation is incorrect. Importantly, the responses to citric acid and acetic acid, which are known to be mediated by Otop1 are unchanged after flecainide, both in behavioral experiments and gustatory nerve recording.

2. As described above, the voltage imaging data is not interpretable without a measurement of the resting voltage (to see if it has changed). In the absence of this experiment, I believe the results are misleading and should be omitted.

3. The patch clamp recording data is not convincing that flecainide acts on Otop1 directly. The decay of the currents has been attributed to acid loading of cells and therefore changes in current decay may be attributed to changes in handling of intracellular pH. I would also like to note that the recovery to baseline of the currents after washoff of the acid stimulus is not as quick as expected, calling into question any conclusions based on kinetics. Instead, I suggest the authors report there is no effect on the magnitude of the currents and omit discussion of the kinetics (unless they are willing to do the hard work of determining if this is due to changes in pH buffering).

4. To test whether flecainide works through Otop1 or even through taste cells, behavior should be measured f Otop1-/- mice or mice missing type III taste cells, which retain acid sensitivity and are available from several laboratories. Without doing these experiments, it would be premature to conclude that flecainide even works through the taste system.

In light of these concerns, and because the data appear to nonetheless be sound, I suggest the authors:

1. rewrite the title to indicate that Otop1 is not likely the target of flecainide. For example, they could use “The antiarrhythmic drug flecainide enhances aversion to HCl in mice”

2. Remove the claim “Unexpectedly, we found that flecainide interacts directly with the sour taste receptor, Otop1” (line 400). There is no evidence for a direct interaction/effect on Otop1.

3. Remove the claim on line 419: “Therefore, we suggest that the primary molecular target of flecainide in the taste system is Otop1 in sour-sensing taste cells”

4. Rewrite the last paragraph: “In conclusion, we found that flecainide enhances sour taste perception via an effect on Otop1 in mouse taste cells.” This is not true.

“Our research provides new insights into the molecular mechanisms of drug-induced taste disorders via Otop1, and may support that Otop1 as a sour taste receptor not only in mice, but also in humans.” Again, I do not believe there is any evidence in support of this statement.

“Our results also imply that the prescription of zinc, Otop1 antagonist, can be potentially used as therapeutic strategy to prevent the taste disfunction and dizziness induced by flecainide” There is no evidence for this in the manuscript. Effect of zinc on Otop1 were previously described.

“research to elucidate the interaction between flecainide and Otop1 at the atomic level

might facilitate the discovery of novel positive/negative allosteric modulators of Otop1.” This is misleading as there is no evidence for a direct effect of flecainide on Otop1.

5. Offer alternative interpretations: The drug may block K+ channels to enhance acid responses (either taste or trigeminal or both). The authors do this on line 453, but it is hidden in the discussion “Thus, flecainide may affect sour taste sensitivity in vivo by influencing the excitability of other molecules such as Kir2.1 as well as Otop1” Please add reference to role of Kir2.1 in sour cells.

Minor:

1. Line 416. Reference for “Recently, it was reported that knockout of Otop1 abolished

sour taste responses from sour-sensing taste receptor cells...” should include Teng et al. 2019.

2. Line 437: “The concentration ranges that induced a sour taste response in mouse CT nerve were very similar to those that activated mOtop1 in HEK293T cells.” First, this data is not novel and thus does not require discussion. Second, the responses in HEK cells are with a pH buffer and thus not comparable to gustatory nerve recording. Leave out.

3. The two-bottle taste preference is not convincing - I would like to see the primary data but it seems it is driven by two outliers in the untreated conditions. Also, the concentration of HCl is much lower that for the brief access, and at a concentration where in the brief access tests there is no difference between the treated and untreated conditions. I would suggest leaving this data out, given the wide variation in behavior in the control group on this assay.

4. In the gustatory nerve recordings, the changes in response to fle are very subtle. I appreciate that the authors show the actual traces, but in Fig 3A it appears there is no change in response to HCl. How was the data measured for Fig 3B?

5. Also please explain if there were any adverse effects of the flecainide

6. Please state why experiments were limited to male mice

7. For the patch clamp and imaging experiments the investigators should state the final pH of the acid solution (5 mM HCl). Presumably there was a buffer present so this will affect the final pH.

8. Why was Otop1 re-cloned from mouse kidney - is there evidence for Otop1 in mouse kidney? Did the sequence match that reported in the literature?
